# Folic acid supplementation acts as a chemopreventive factor in tumorigenesis of hepatocellular carcinoma by inducing H3K9Me2-dependent transcriptional repression of LCN2

**DOI:** 10.18632/oncotarget.27136

**Published:** 2021-02-16

**Authors:** Yin-Ling Zhang, Geng Xue, Hui Miao, Chuan-Chuan Zhou, Shu-Han Sun, Yi Zhang

**Affiliations:** ^1^ Department of Medical Genetics, College of Basic Medical Sciences, Second Military Medical University, Shanghai, 200433, China; ^2^ Department of Clinical Genetics Division, Changzheng Hospital, Second Military Medical University, Shanghai, 200003, China

**Keywords:** folic acid, hepatocarcinogenesis, chemoprevention, LCN2, H3K9Me2

## Abstract

The effects and mechanisms of folic acid (FA) as a chemopreventive agent for tumorigenesis of hepatocellular carcinoma (HCC) remain unclear. In this study, the QSG-7701, a human normal liver cell line, was cultured in different FA levels (High, Normal or No) for 6 months. Then, the biological characteristics, the expression of main stem cell-like genes or epithelial-mesenchymal transition (EMT) related genes and the tumorigenicity *in vivo* of cells cultured in different treatment groups were detected. Our results showed that No FA improved the malignant transformation of cells but High FA depressed the malignant transformation. Meanwhile, cells in different treatment groups were mapped by transcriptome sequencing. Then the relativity of increased LCN2 and decreased FA level was identified and confirmed *in vitro* and vivo. We also revealed that intracellular control of LCN2 would recover the effects of FA on cell proliferation, cell cycle and tumor formation *in vitro* and vivo. Finally, our studies displayed that increased FA level induced the down-regulation of LCN2 not by DNA hypermethylation of LCN2 promoter but by promoting the level of histone H3 lysine 9 di-methylation (H3K9Me2) in LCN2 promoter. In conclusion, our studies disclosed the chemopreventive effect of FA supplementation on hepatocarcinogenesis, which partial attributed to the inhibition of LCN2 by regulating histone methylation in promoter. Our results provide a potential mechanism of the chemoprevention of FA supplementation on tumorigenesis of HCC and may be helpful in developing treatment target against HCC.

## INTRODUCTION

Hepatocellular carcinoma (HCC) is the primary type of liver cancer and the second most common cause of cancer-related death worldwide [1, 2]. Patients with the disease often undergo treatments involving resection, microwave ablation, and liver transplantation, which offer the only chance of a cure. However, only one-third of patients are suitable for the above treatments [3]. The World Health Organization (WHO) has recommended hepatitis B vaccine immunization for infants and children to interrupt the possible transmission routes and the treatment of eligible patients with antiviral agents. However, these measures do not offer the possibility of completely curing the disease [4]. Thus, other prevention strategies are needed.

Folic acid (Folate, FA) is a water-soluble vitamin that humans cannot produce and must receive by dietary intake [5]. FA plays a major role in two 1-carbon transfer pathways, including biological methylation reaction and nucleotide biosynthesis. Previous studies have proved that the deficiency of FA would result in inadequate DNA damage repair and impairs cellular proliferation by inducing aberrant DNA methylation, which increase the risk of various tumors [6–8]. Moreover, some studies also revealed that the supplementation of FA would significantly reduce inflammation and prevent the tumorigenesis of tumors by increasing global DNA methylation [5, 9, 10]. However, what makes people more interesting was that some previous studies showed that FA supplementation would significantly decrease the risk of cancer in a mouse model without precancerous lesions but other studies revealed that such supplementation would also promote the development of precancerous lesions by providing methyl units to rapidly replicating transformed cells and inducing accelerated cell proliferation [10, 11]. Although above contradictory phenomena was disturbing, epidemiological studies have shown that individuals without a long-term folate supplementation had a 2-fold greater incidence of colonic neoplasia than people who had taken folate supplementation [5, 12].

Previous epidemiological studies displayed the widespread FA deficiency in China, especially in North China [13]. Approximately 32% to 35% of women in North China showed low serum and red blood cell FA levels, which induced highest incidence rate of neonatal neural tube defects (NTDs) around the world and daily supplementation with 0.4 mg FA in early stage of pregnancy would reduce the risk of NTDs by 85% [14]. It is well-known that around 50% of new cases of HCC now occur in China [15] and it was proved that high serum folate levels are inversely associated with liver damage and HCC [16]. However, the definitely relationship between FA with HCC and the exact role of FA in tumorigenesis of HCC are all still unclear.

Therefore, the biological characteristics and malignant transformation of human normal liver cell line QSG-7701, cultured in high, normal or no levels of FA for 6 months, were all detected *in vitro* and vivo. Then, one important oncogene, LCN2 [17], were screened and identified by transcriptome sequencing and the regulation mechanism of FA on LCN2 were also characterized *in vitro* and vivo. Finally, the chemoprevention of FA supplementation on hepatocarcinogenesis by increasing the level of H3K9Me2 in LCN2 promoter and depressing its expression was approved in our study.

## RESULTS

### The effect of FA deficiency or supplementation on malignant transformation of QSG-7701 cells *in vitro* and *in vivo*

#### The effect of FA deficiency or supplementation on cell proliferation and cell cycle

**Figure 1:**
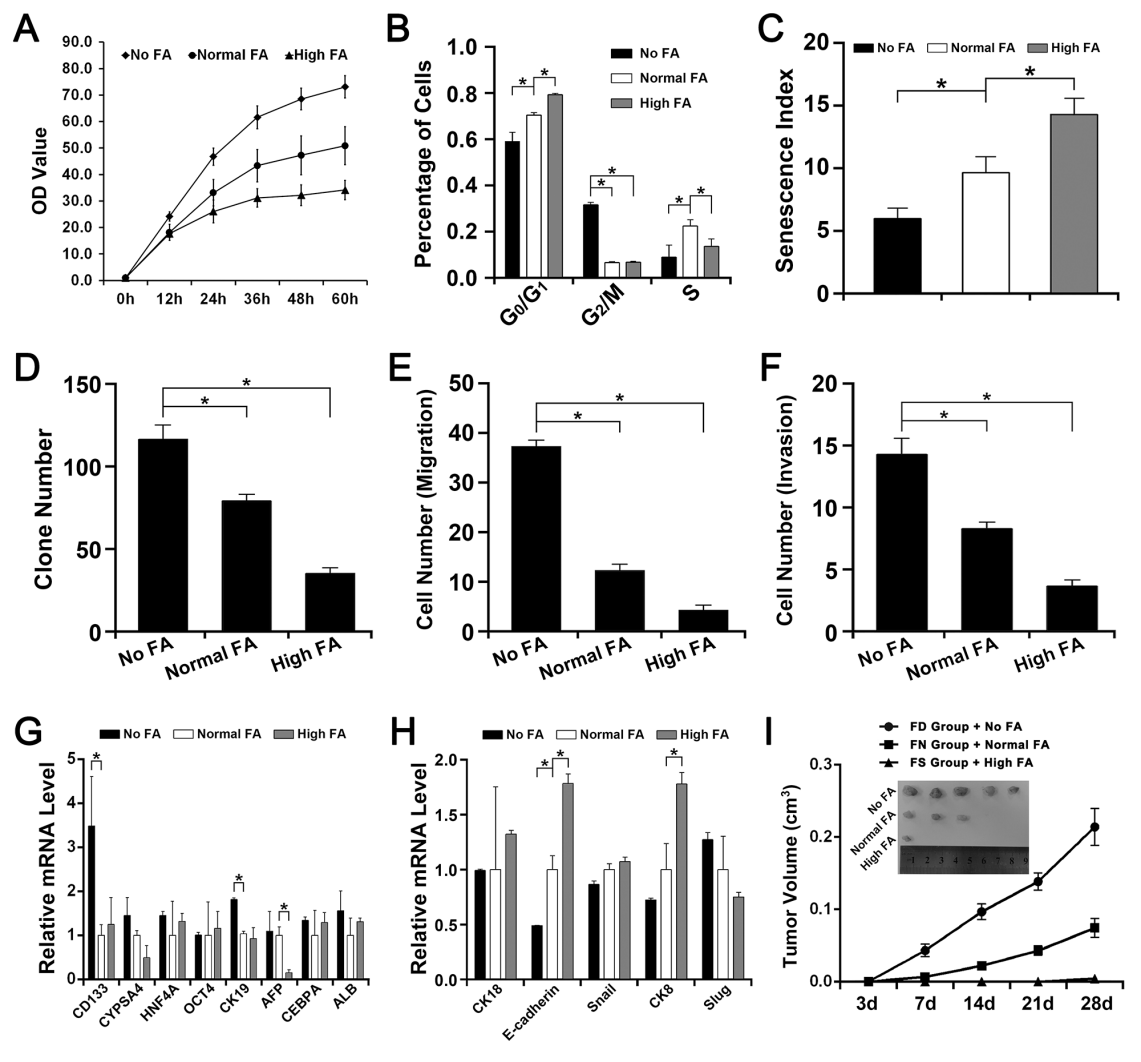
The effect of FA deficiency or supplementation on QSG-7701 cells *in vitro* and *in vivo* The QSG-7701 cell cultured with 0.0mg/L FA was represented as No FA. The QSG-7701 cell cultured with 4.0mg/L FA was represented as Normal FA. The QSG-7701 cell cultured with 40.0mg/L FA was represented as High FA. The FA-deficiency nude mice fed with 0mg folate/kg diet was represented as FD group. The FA-normal nude mice fed with 2mg folate/kg diet was represented as FN group. The FA-supplementation nude mice fed with 8mg folate/kg diet was represented as FS group. **(A)** CCK-8 assay revealed that the proliferation rate of Normal FA was slower than No FA but faster than High FA (^*^ *P*<0.001). **(B)** Flow cytometry assay revealed that more High FA were in G_0_/G_1_ phases and more No FA were in G_2_/M phases (^*^ *P*<0.001). **(C)** β-galactosidase staining assay revealed that the senescence index of Normal FA was higher than No FA but lower than High FA (^*^ *P*<0.001). **(D)** Colony formation assay revealed that the efficiency of Normal FA was lower than No FA but higher than High FA (^*^ *P*<0.001). **(E)** Transwell assay revealed that the migrated cell number of Normal FA was smaller than No FA but larger than High FA (^*^ *P*<0.001). **(F)** Transwell assay revealed that the invasive cell number of Normal FA was smaller than No FA but larger than High FA (^*^ *P*<0.001). **(G)** The mRNA level of several stem cell-like genes in No FA, Normal FA and High FA (^*^ *P*<0.001). **(H)** The mRNA level of several EMT-related genes in No FA, Normal FA and High FA (^*^ *P*<0.001). **(I)** Tumor formation assay in nude mouse revealed that the tumor growth rate of FN group with Normal FA was slower than FD group with No FA but faster than FS group with High FA and the tumor volume of FN group with Normal FA was smaller than FD group with No FA but bigger than FS group with High FA (^*^ *P*<0.001).

With the extension of treatment, CCK-8 assay revealed that FA deficiency (No FA) improved the proliferation of QSG-7701 cells and FA supplementation (High FA) depressed the proliferation of QSG-7701 cells (Figure 1A). Our results were consistent with previous studies that showed an increase in cell proliferation during hepatocarcinogenesis [18].

As summarized in Figure 1B, more QSG-7701 cells with High FA were in G_0_/G_1_ phases than cells with Normal FA but less QSG-7701 cells with High FA were in S phases than cells with Normal FA. More QSG-7701 cells with No FA were in G_2_/M phases than cells with Normal FA or High FA but less QSG-7701 cells with No FA were in G_0_/G_1_ phases and S phases than cells with Normal FA.

#### The effect of FA deficiency or supplementation on cell senescence

In our study, β-galactosidase staining assay revealed that more QSG-7701 cells with High FA showed a positive staining of β-galactosidase than cells with Normal FA but less QSG-7701 cells with No FA showed a positive staining of β-galactosidase than cells with Normal FA (Figure 1C).

#### The effect of FA deficiency or supplementation on cell migration, invasion and colony formation

Transwell assay and colony formation assay revealed that FA deficiency (No FA) improved the migration, invasion and colony formation of QSG-7701 cells and FA supplementation (High FA) depressed the migration, invasion and colony formation of QSG-7701 cells (Figure 1D, 1E, 1F).

#### The effect of FA deficiency or supplementation on the expression of some stem cell-like genes

The expressions of CD133 and CK19 in QSG-7701 cells with No FA were both higher than in cells with Normal FA and in cells with High FA. The expression of CYPSA4 in QSG-7701 cells with No FA was higher than in cells with High FA. The expression of AFP QSG-7701 cells with High FA was lower than in cells with Normal FA and in cells with No FA (Figure 1G).

#### The effect of FA deficiency or supplementation on the expression of some EMT-related genes

The expression of E-cadherin in QSG-7701cells with No FA was lower than in cells with Normal FA and in cells with High FA. The expressions of CK8 and CK18 in QSG-7701cells with No FA were both lower than in cells with High FA. The expression of Slug in QSG-7701cells with No FA was higher than in cells with High FA. The expressions of E-cadherin and CK8 in QSG-7701cells with High FA were both higher than in cells with Normal FA (Figure 1H).

#### The effect of FA deficiency or supplementation on tumor formation in nude mouse assay (xenografts)

Less FS group mice injected with High FA QSG-7701cells (1 of 5) developed a small tumor. Some FN group mice injected with Normal FA QSG-7701cells (3 of 5) developed smaller tumors. All FD group mice injected with No FA QSG-7701cells (5 of 5) developed large tumors (Figure 1I). The tumor volume of FD group was larger than the other two groups and the tumor growth of FD group was also faster than the other two groups (Figure 1I).

### Transcriptome sequencing and validation by quantitative real-time (Q-RT) PCR

A global view of the difference of transcript expression in QSG-7701cells maintained in No FA, Normal FA or High FA was then detected by transcriptome sequencing and a total of 16774 RNAs were detected. Excluding these differential RNAs that did not conform to the selection principles (a fold change ≥ 1.5 and a p-value ≤ 0.05) or was not linearly related to the folate concentration, there were 391 differential RNAs were enrolled in further assays. Among these differential RNAs, the levels of 115 RNAs were up-regulated and the levels of another 276 RNAs were down-regulated with the decrease of folic acid concentration.

**Figure 2:**
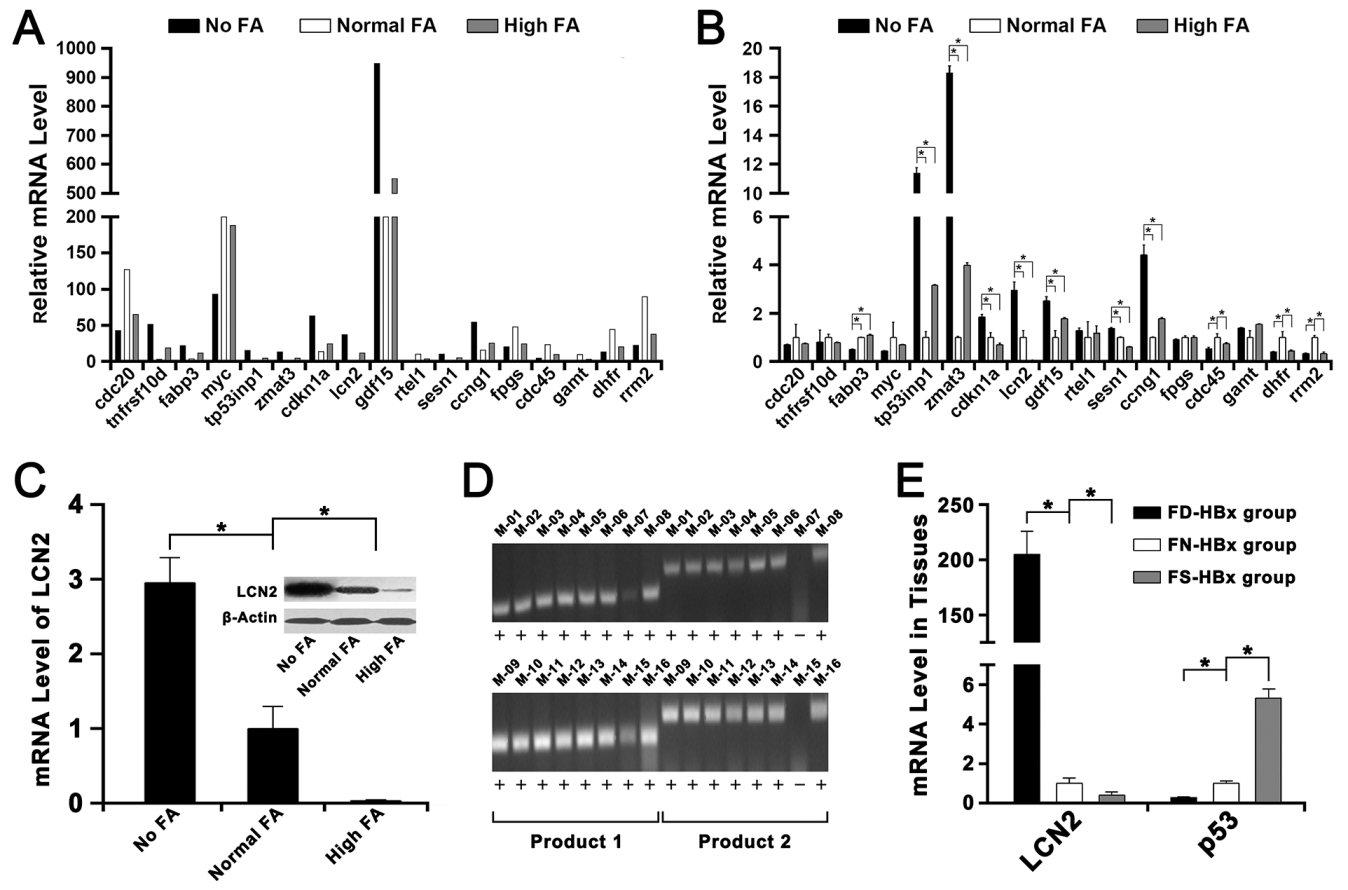
Transcriptome sequencing and validation of the RNA-seq data by Q-RT PCR The FA-deficiency HBx transgenic mice fed with 0mg folate/kg diet was represented as FD-HBx group. The FA-normal HBx transgenic mice fed with 2mg folate/kg diet was represented as FN-HBx group. The FA-supplementation HBx transgenic mice fed with 8mg folate/kg diet was represented as FS-HBx group. **(A)** The mRNA level of several genes in No FA, Normal FA and High FA detected by transcriptome sequencing. **(B)** The mRNA level of these genes in No FA, Normal FA and High FA detected by Q-RT PCR (^*^ *P*<0.001). **(C)** The mRNA and protein levels of LCN2 in Normal FA were both lower than in No FA but higher than in High FA (^*^ *P*<0.001). **(D)** The HBx-positive transgenic mice were identified by PCR and agarose gel electrophoresis. Mice that had both two positive results (product 1 & product 2) were defined as HBx positive. **(E)** The mRNA level of LCN2 in FN-HBx group was lower than in FD-HBx group but higher than in FS-HBx group; however, the mRNA level of p53 in FN-HBx group was higher than in FD-HBx group but lower than in FS-HBx group (^*^ *P*<0.001).

GO analysis showed the top five most significant gene functions of 391 differential RNAs were cell cycle arrest, negative regulation of phosphorylation, response to organic nitrogen, single-stranded DNA specific 5’-3’ exodeoxyribonuclease activity and positive regulation of metanephric cap mesenchymal cell proliferation (Supplementary Figure 1A). Pathway analysis showed the top five most significant pathways involved were p53 signaling pathway, bladder cancer, chronic myeloid leukemia, small cell lung cancer and ErbB signaling pathway (Supplementary Figure 1B). Some affected genes which were closely related to the above functions and pathways were selected to validate the results of high throughput sequencing by Q-RT PCR. Our results revealed that the data of nearly all genes detected by RNA-seq (Figure 2A) were consistent with the expression profiles detected by Q-RT PCR (Figure 2B).

Among these selected genes, the LCN2, which was an important oncogene and proved to be controlled by DNA methylation in tumorigenesis [19, 20], was selected as the target gene for further studies because of its significantly abnormal expression induced by FA deficiency or supplementation (Figure 2C). Then, the mRNA level of LCN2 and p53 were detected in HBx-positive mice (Figure 2D). The mRNA level of LCN2 in liver tissues of FA-deficiency HBx-positive mice (FD-HBx group) was higher than FA-normal HBx-positive mice (FN-HBx group) and the mRNA level of LCN2 in liver tissues of FA-supplementation HBx-positive mice (FS-HBx group) was lower than FA-normal HBx-positive mice (Figure 2E). The mRNA level of p53 in liver tissues of FA-deficiency HBx-positive mice (FD-HBx group) was lower than FA-normal HBx-positive mice (FN-HBx group) and the mRNA level of p53 in liver tissues of FA-supplementation HBx-positive mice (FS-HBx group) was higher than FA-normal HBx-positive mice (Figure 2E).

### The effects of LCN2 on FA deficiency induced malignant transformation of QSG-7701 cells

#### The cellular localization of LCN2 in QSG-7701 cells

**Figure 3:**
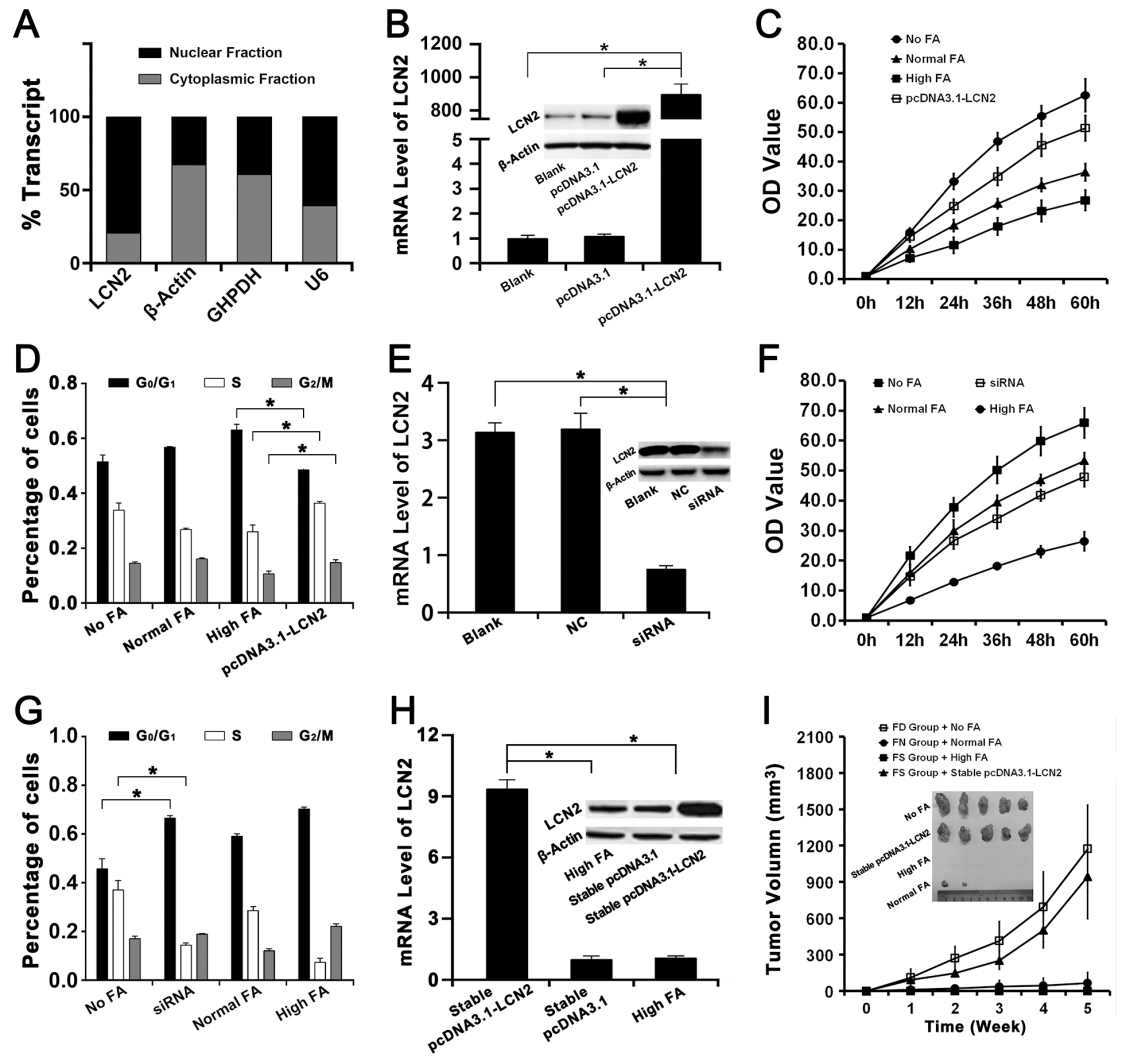
The effects of LCN2 on FA deficiency induced hepatocarcinogenesis and the chemoprevention of FA supplementation The High FA transfected with pcDNA3.1-LCN2, empty pcDNA3.1 or nothing were represented as pcDNA3.1-LCN2, pcDNA3.1 or Blank respectively. The No FA transfected with siRNA of LCN2, negative control or nothing were represented as siRNA, NC or Blank respectively. The High FA stably transfected with pcDNA3.1-LCN2 or empty pcDNA3.1 were represented as stable pcDNA3.1-LCN2 or stable pcDNA3.1. **(A)** Cellular localization assay revealed that LCN2 was primarily localized in the nucleus of QSG-7701 cells. **(B)** The mRNA and protein levels of LCN2 in High FA transfected with pcDNA3.1-LCN2 were both higher than in High FA transfected with pcDNA3.1 and High FA transfected with nothing (^*^ *P*<0.001). **(C)** CCK-8 assay revealed that the proliferation rate of High FA transfected with pcDNA3.1-LCN2 was faster than High FA and Normal FA but slower than No FA (^*^ *P*<0.001). **(D)** Flow cytometry assay revealed that the percentage of High FA transfected with pcDNA3.1-LCN2 in G_0_/G_1_ phases was lower than High FA but the percentage of High FA transfected with pcDNA3.1-LCN2 in S or G_2_/M phases were higher than High FA (^*^ *P*<0.001). **(E)** The mRNA and protein levels of LCN2 in No FA transfected with siRNA of LCN2 were both lower than in No FA transfected with negative control and No FA transfected with nothing (^*^ *P*<0.001). **(F)** CCK-8 assay revealed that the proliferation rate of No FA transfected with siRNA of LCN2 was lower than No FA and Normal FA but faster than High FA (^*^ *P*<0.001). **(G)** Flow cytometry assay revealed that the percentage of No FA transfected with siRNA of LCN2 in G_0_/G_1_ phases was higher than No FA but the percentage of No FA transfected with siRNA of LCN2 in S phase were lower than No FA (^*^ *P*<0.001). **(H)** The mRNA and protein levels of LCN2 in stable pcDNA3.1-LCN2 were both higher than in stable pcDNA3.1 and High FA (^*^ *P*<0.001). **(I)** Tumor formation assay in nude mouse revealed that the tumor growth rate of FS group with stable pcDNA3.1-LCN2 was faster than FS group with High FA and FN group with Normal FA. The tumor volume of FS group with stable pcDNA3.1-LCN2 was also bigger than FS group with High FA and FN group with Normal FA (^*^ *P*<0.001).

The mRNA of LCN2 in cytoplasm and nucleus of QSG-7701 cells were isolated respectively and normalized against β-actin, GAPDH and U6 to detect the cellular localization of LCN2 (Figure 3A). Our results revealed that LCN2 was primarily localized in the nucleus of QSG-7701 cells.

#### Up-regulation of LCN2 recovered the effects of FA supplementation on cell proliferation and cell cycle

The expression of LCN2 in QSG-7701 cells with High FA was up-regulated by transfecting with the plasmid pcDNA3.1-LCN2 (Figure 3B). Then the up-regulation of LCN2 improved cell proliferation of QSG-7701 cells with High FA (Figure 3C) and moved cell cycle progression beyond the G_1_/S transition in QSG-7701 cells with High FA (Figure 3D).

#### Down-regulation of LCN2 recovered the effects of FA deficiency on cell proliferation and cell cycle

The expression of LCN2 in QSG-7701 cells with Low FA was down-regulated by transfecting with the siRNA for LCN2 (Figure 3E). Then the down-regulation of LCN2 repressed cell proliferation of QSG-7701 cells with Low FA (Figure 3F) and arrested cell cycle progression at G_1_/S phase in QSG-7701 cells with Low FA (Figure 3G).

#### Stable up-regulation of LCN2 improved tumor formation of QSG-7701 cells with High FA *in vivo*

As summarized in Figure 3H, the expression level of LCN2 in stable transfectants of pcDNA3.1-LCN2 (QSG-7701 cells with High FA & pcDNA3.1-LCN2) was higher than stable transfectants of pcDNA3.1 (QSG-7701 cells with High FA & pcDNA3.1) and then stable transfectants of pcDNA3.1-LCN2 or pcDNA3.1 were injected into FS group nude mice respectively. Our results revealed the tumor size in mice of FS group injected with stable transfectants of pcDNA3.1-LCN2 were much larger than mice of FS group injected with stable transfectants of pcDNA3.1 (Figure 3I) and the tumor growth rate of FS group injected with stable transfectants of pcDNA3.1-LCN2 was similar to FD group injected with QSG-7701 cells with No FA (Figure 3I).

### Effect of FA deficiency or supplementation on DNA methylation in the promoter of LCN2

Because of the crucial of FA in DNA methylation, the DNA methylation levels of CpG islands in the promoter of LCN2 were detected by bisulfite genomic sequencing assay. The region in the promoter of LCN2 (-238 ~ -19 upstream of transcriptional start site), which was selected to detect in our study, has already been proved to contain multiple key transcription initiation elements, including TATA box, C/EBP-α&β binding sites, AP-1 binding site, NF-γ binding site and NF-κB binding site, and the changes of DNA methylation level in this region were crucial for the transcription of LCN2 in tumorigenesis [20].

**Figure 4:**
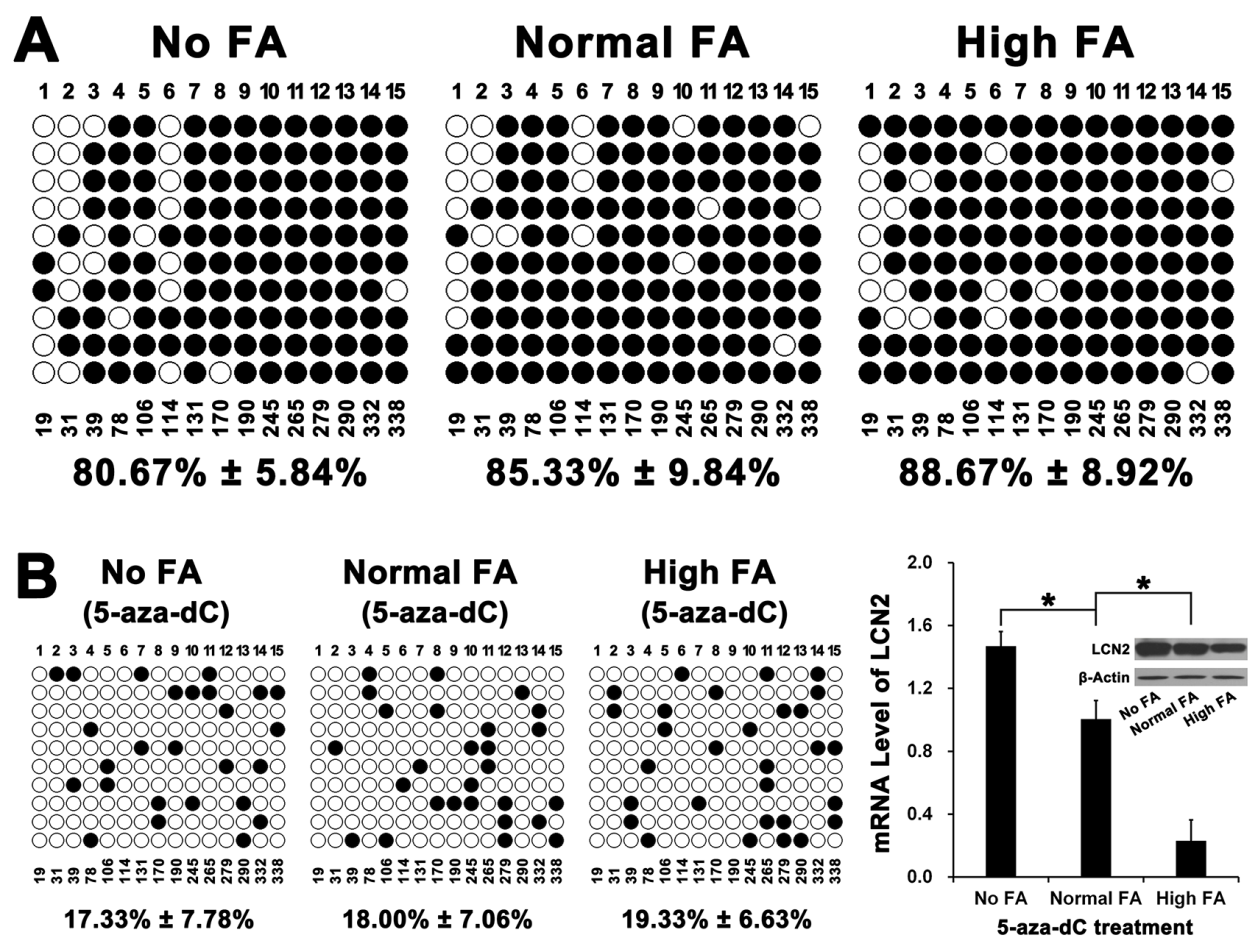
The effect of FA on DNA methylation of CpG island in the promoter of LCN2 **(A)** The CpG island detected in our study locates in -238 ~ -19 upstream of transcriptional start site of LCN2 and includes 15 CpG sites. Bisulfite genomic sequencing assay revealed that there was no significant difference in DNA methylation rates among No FA, Normal FA and High FA (*P*>0.001). **(B)** After treatment of 5-aza-dC for 71 hours, bisulfite genomic sequencing assay revealed that the DNA methylation rates of LCN2 promoter in cells cultured with No FA, Normal FA and High FA were all reduced. However, there was also no significant difference in DNA methylation rates among No FA, Normal FA and High FA (*P*>0.001) and the mRNA & protein levels of LCN2 in Normal FA were also both lower than in No FA but higher than in High FA (^*^ *P*<0.001).

Our results revealed that, in relation to QSG-7701 cells with Normal FA, there were no significant difference in DNA methylation level of CpG island in the promoter of LCN2 in QSG-7701 cells with No FA or QSG-7701 cells with High FA (Figure 4A). Moreover, the treatment of 5-Aza-2’-deoxycytidine (5-aza-dC, an important DNA methyltransferase inhibitor [20]) down-regulated the level of DNA methylation of CpG island in the promoter of LCN2 in QSG-7701 cells with No, Normal and High FA (Figure 4B). However, after 5-aza-dC treatment, the expression LCN2 in QSG-7701 cells with High FA was still lower than QSG-7701 cells with Normal or No FA, even if there were no significant difference in DNA methylation level of CpG island in the promoter of LCN2 in QSG-7701 cells with No, Normal and High FA (Figure 4B).

### Effect of FA deficiency or supplementation on histone methylation in the promoter of LCN2

**Figure 5:**
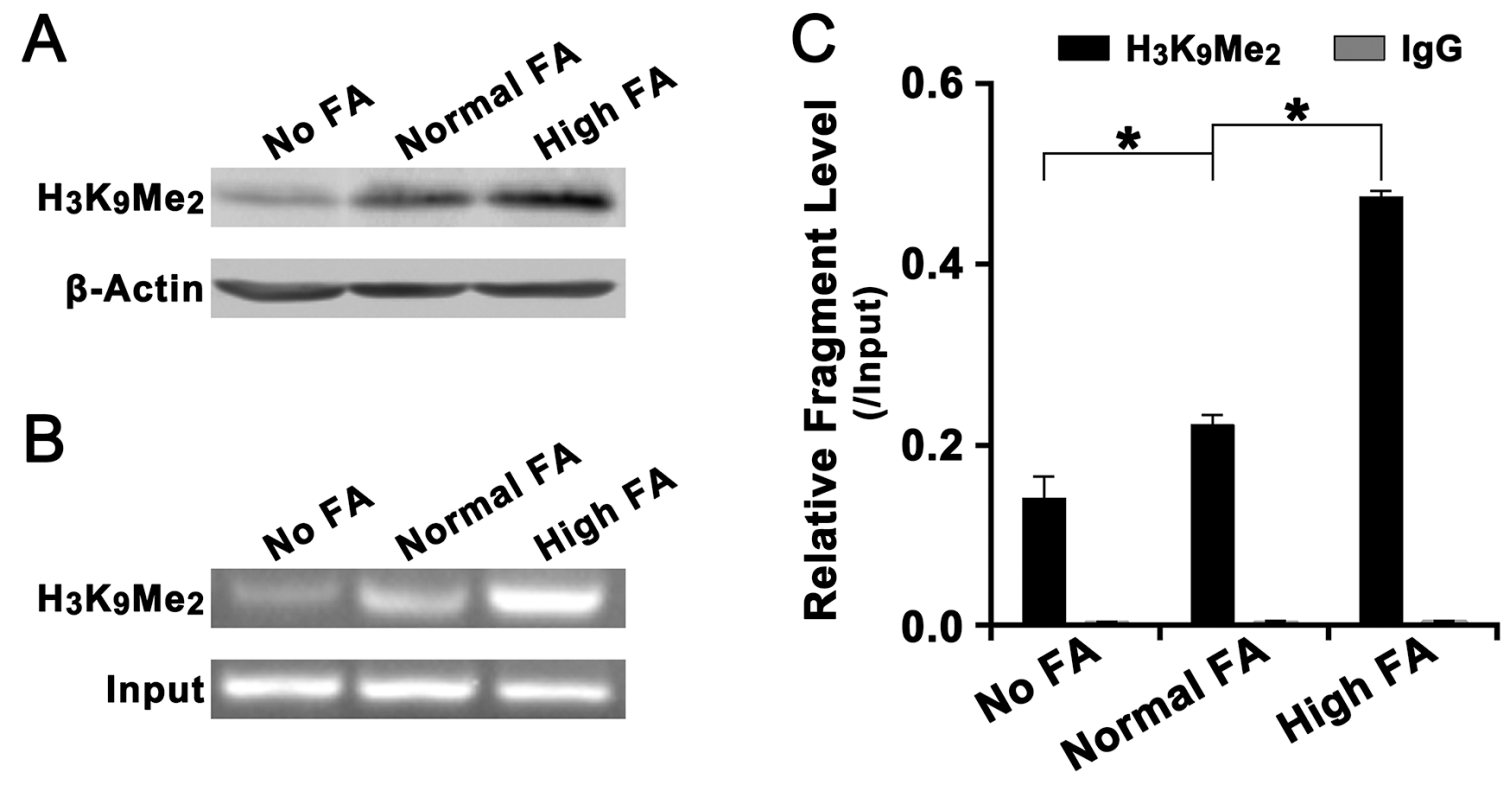
Effect of FA on the level of histone H3K4Me2 in the promoter of LCN2 **(A)** Western blot assay revealed that, relative to β-actin, the overall level of histone H3K4Me2 in Normal FA was higher than in No FA but lower than in High FA. **(B)** Agarose gel electrophoresis assay on LCN2 promoter specific ChIP-PCR products revealed that, relative to input, the level of ChIP-PCR products in Normal FA was higher was higher than in No FA but lower than in High FA. **(C)** Statistical analysis revealed that, relative to input, the level of histone H3K4Me2 in the promoter of LCN2 in Normal FA was higher than in No FA but lower than in High FA (^*^ *P*<0.001).

Since FA deficiency had no effect on DNA methylation of LCN2 promoter, we further investigated the effect of FA deficiency or supplementation on histone methylation of LCN2 promoter. As a repressive transcriptional status, the decrease of histone H3K9 methylation level would induce the elevation of many oncogenes during tumorigenesis [21]. Our results revealed that the level of histone H3K9 di-methylation (H3K9Me2) in the promoter of LCN2 was significant down-regulated in QSG-7701 cells with No FA but the level of histone H3K9 di-methylation (H3K9Me2) was significant up-regulated in QSG-7701 cells with High FA (Figure 5A). ChIP assay on the 1,000 bp upstream of transcriptional start site (TSS) of LCN2, which contained predicted H3K9Me2 binding sites, revealed a significant enhancement of binding of H3K9Me2 to the promoter of LCN2 in QSG-7701 cells with High FA compared with QSG-7701 cells with Normal FA and No FA (Figure 5B, 5C).

### Up-regulation of LCN2 and decline of FA level in tumor tissues of HCC patients

**Figure 6:**
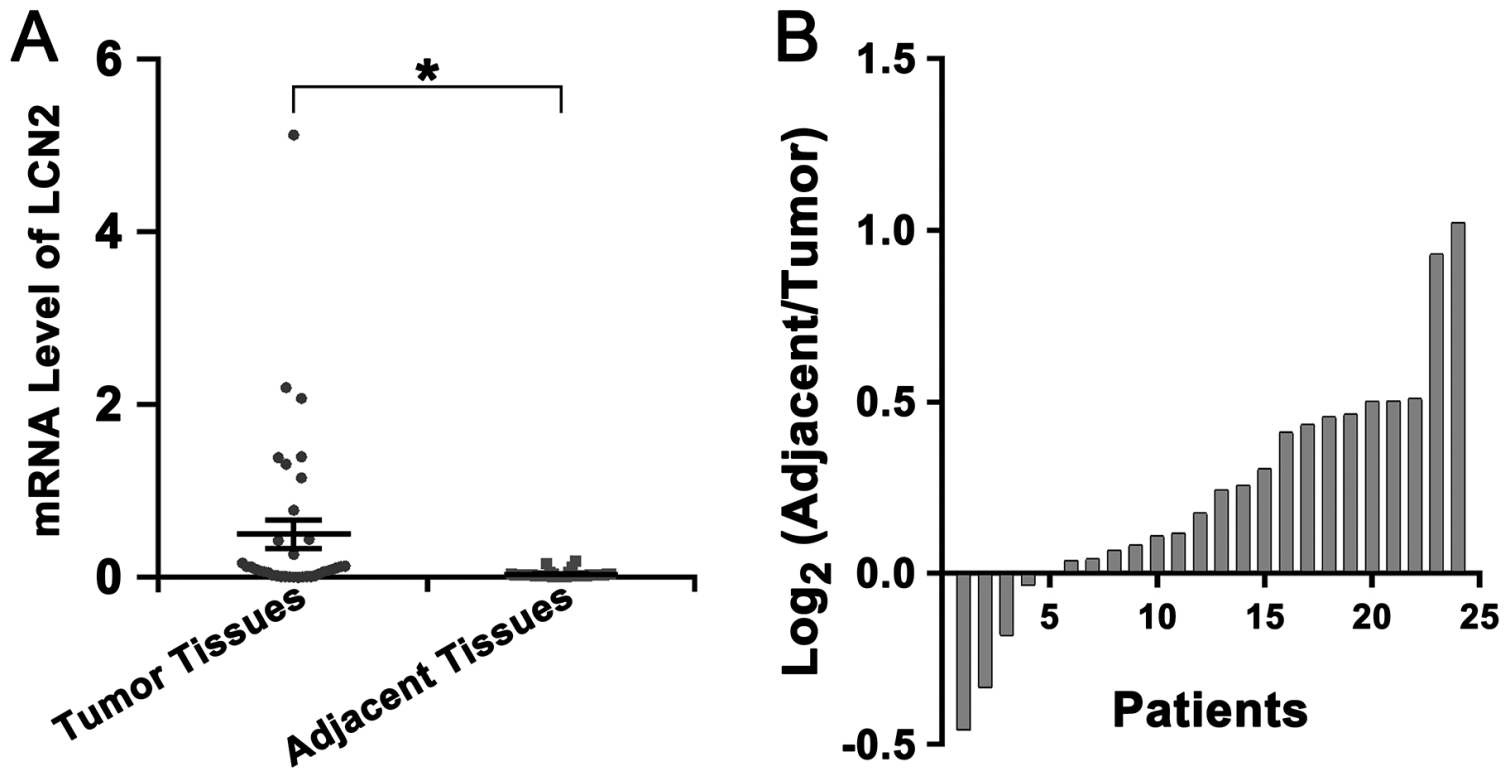
Up-regulation of LCN2 and decline of FA level in tumor tissues of HCC patients **(A)** The mRNA level of LCN2 in tumor tissues was higher than in adjacent tissues (^*^ *P*<0.001). **(B)** Most patients (19 of 24) showed a lower FA level in tumor tissues than in adjacent tissues (The ratio of the level of FA in adjacent tissue to the level of FA in tumor tissues was greater than 1).

The expression of LCN2 and the level of FA in tumor tissues and corresponding adjacent tissues of HCC patients were also detected. Our results revealed that the level of LCN2 in tumor tissues was higher than in corresponding adjacent tissues (Figure 6A) but the level of FA in tumor tissues was lower than in adjacent tissues (Figure 6B). However, we failed to find a confirmed association between the level of FA and the degree of lymph node metastasis, infiltration, or clinical HCC stage.

## DISCUSSION

As we all know, malignant proliferation of cells was associated with tumorigenesis and considered to be an early event of cancer [22, 23]. Our data displayed that FA supplementation depressed cell proliferation and promoted cell senescence. Moreover, because the arrest of cell cycle would lead to the decrease of cell proliferation rate [24] and the central feature of senescent cells is cell cycle arrest [25]. In our studies, we also found that FA supplementation induced cell cycle arrest at the G_0_/G_1_ phase and reduced the numbers of cells distributed in S phase. Our results indicated that the effects of FA supplementation on cell cycle arrest may be one of the most important aspects of the chemoprevention of FA supplementation on tumorigenesis. Previous studies have proved the key feature of the early and later stages of cancer is the inactivation of the differentiation marker E-cadherin and the inhibited expression of E-cadherin is an indicative of increased cancer risk [26, 27]. In our studies, we found that FA supplementation resulted in an enhanced E-cadherin expression but FA deficiency resulted in a reduced E-cadherin expression. And FA supplementation repressed the migration and invasion of QSG-7701 cells and FA deficiency improved the migration and invasion of cells. It deemed that long time FA deficiency lead to a loss of E-cadherin expression and thus an altered cell phenotype on metastasis.

All the time, the relationship of FA deficiency and tumourgenesis is always attractive but controversial. A lot of studies believe that FA deficiency can be a risk factor for tumor [9–11, 28]. However, the exact relationship and mechanism are not clear. In our studies, we found that FA deficiency improved the malignant transformation and tumor formation of QSG-7701 cells *in vitro* and *in vivo*. Especially in nude mouse, FA deficiency improved tumor probability and tumor size compared to FA normal and FA supplementation. In addition, we also found that the level of FA in tumor tissues of HCC patients was lower than in adjacent tissues. Once again our results indicated that FA deficiency remains a risk factor of tumorigenesis. Interestingly, we failed to find a confirmed association between FA level and the degree of lymph node metastasis, infiltration, or clinical HCC stage. In our opinion, FA deficiency might confer a high risk of tumor initiation but not progression.

As an important oncogene, LCN2 was reported to promote invasion through its physical association with matrix metalloproteinase-9, thereby contributing to the tumorigenicity of several human tumors [17, 19, 29–31]. In our studies, we disclosed that up-regulation of LCN2 could recover the chemoprevention of FA supplementation on tumorigenesis. Our results also indicated that LCN2 played an important role in facilitating FA deficiency induced tumorigenesis of HCC. In addition, another study proved that the mechanism of cell cycle arrest at the G_0_/G_1_ phase by LCN2 knockdown involved a decrease in cell-cycle related proteins (cyclin D1, P21, and phosphor-p53) [24]. Our results also revealed that LCN2 promoted cell proliferation by facilitating cell cycle progression during the course of tumorigenesis. In our opinion, LCN2 may be a potential therapeutic target in clinical treatment of HCC.

It is well known that FA through its role in one carbon metabolism is crucial for DNA methylation [32]. In normal cells, DNA methylation is related to oncogene repression, which is crucial for cell proliferation, differentiation, and chromosomal integrity. Alterations in DNA methylation patterns play an important role in many malignancies and have been related to cancer initiation [8–12]. However, in this study we failed to find DNA methylation alterations induced by FA deficiency in the promoter of LCN2 and the reduced expression of LCN2 induced by FA supplementation may not be caused by regulating DNA methylation. Previous studies indicated that histone modifications, such as methylation or acetylation, also played an important role in a variety of cellular processes, gene functions or tumorigenesis [33]. Histone H3K9 methylation is known to be associated with a repressive transcriptional status and the decrease in the level of histone H3K9 methylation denotes poor prognosis in cancer patients [21, 34]. Our results also disclosed that, by directly improving the level of H3K9Me2 in the promoter of LCN2, FA supplementation suppress the expression of LCN2 and develop chemoprevention on hepatocarcinogenesis.

The association between hepatitis B virus (HBV), the HBx gene of HBV and HCC has been well-known [35]. To confirm the FA chemoprevention on HBV induced hepatocarcinogenesis, we also detected the effects of FA deficiency or supplementation on LCN2 and p53 in HBx transgenic mice. Interestingly, our results displayed that the level of LCN2 was down-regulated but the level of p53 was up-regulated in HBx transgenic mice with FA supplementation for 32 weeks. Previous studies proved that approximately 60% of HBx transgenic mice develop HCC after 18 months, which is shorter than the course of human HCC that develops from chronic HBV infection, and p53 is a developmental repressor of AFP transcription in normal liver tissues [36, 37]. It seemed that FA may be used to HBV infected patients in chemoprevention of HCC.

In conclusion, our study revealed that FA supplementation repressed the tumorigenesis of HCC by enhancing the level of histone H3K9Me2 in the promoter of LCN2 and then repressing the expression of LCN2. Our results provide a potential mechanism of the chemoprevention of FA supplementation on hepatocarcinogenesis and may be helpful in developing an effective early diagnostic marker or treatment target against HCC.

## MATERIALS AND METHODS

### Samples and treatments

The human normal liver cell line QSG-7701 was obtained from the American Type Culture Collection (VA, USA) and maintained in different FA (Sigma-Aldrich, MO, USA) level as follows: No FA (0.0 mg/L), Normal FA (4.0 mg/L) and High FA (40.0 mg/L). The cells were cultured at 37°C containing 5% CO_2_ in Dulbecco’s modified Eagle’s medium (FA deficiency, D2429, Sigma-Aldrich) with 15% clear constituent KnockOut™ Serum Replacement (Life Technologies, CA, USA).

QSG-7701 cells in different FA treatment groups were also were seeded in 10 cm culture dishes. After overnight incubation, the culture medium was replaced with fresh medium containing with 1 μM 5-aza-dC (A3656, Sigma) or DMSO (negative control), and further incubated for 72 hours. At the end of treatment, cells were harvested for further studies.

Male athymic nude mice (5 weeks old) were purchased from the Chinese Academy of Sciences (Shanghai, China) and maintained in a pathogen-free facility. Animal welfare and experimental procedures were performed strictly according to the Guide for the Care and Use of Laboratory Animals published by the United States National Institutes of Health.

HBx transgenic mice used in this study were constructed by the Model Animal Research Centre of Nanjing University (Nanjing, China). Animal welfare and experimental procedures were performed also strictly according to the Guide for the Care and Use of Laboratory Animals published by the United States National Institutes of Health.

A total of 24 consecutive patients (17 males and 7 females) with primary HCC were enrolled in our studies. Written informed consent was obtained prior to the study. The research protocol and consent form were approved by the Ethics Review Committee of Second Military Medical University. All tumor tissues were taken and confirmed by three experienced pathologists. Corresponding adjacent tissues from patients were also taken synchronously. Tissues were frozen in liquid nitrogen immediately after surgical resection.

### Plasmids and small interfering RNA (siRNA)

The eukaryotic expression vector of LCN2 (pcDNA3.1-LCN2) was constructed in our laboratory and all primes are listed in Supplementary Table 1. The siRNA for LCN2 and negative control (NC) were purchased from GenePharma (Shanghai, China) and the sequence were listed in Supplementary Table 1.

### Antibodies

The monoclonal antibodies for LCN2 (ab187370) and H3K9Me2 (ab1220) were all purchased from Abcam (UK). The monoclonal antibodies for β-actin (A5441) and the secondary antibody (Anti-Mouse IgG, SAB3701044) were all purchased from Sigma-Aldrich.

### Cellular proliferation assay

Cell proliferation assays were performed in 96-well plates (4×10^3^ cells/well) using a Cell Counting Kit-8 (CCK-8, Dojjndo, Japan) according to the instructions with a microplate reader (BIO-RAD, USA). The absorbance in each well at 450 nm was measured at 0, 12, 24, 36, 48, and 72 h. Then, the relative values at each time point were obtained based on the ratio to background value measured form wells with no cells but CCK-8. For each case, three independent experiments were repeated.

### Cell cycle assay

Cell cycle assay was performed by flow cytometry with a FACSCalibur (BD Biosciences, San Jose, CA, USA). The cells were fixed with 70% ethanol overnight and then stained with 20 μg/ml PI containing 20 μg/ml of RNase for 2 h. Normal FA cells was used as the negative control to exclude the influence of autofluorescence and to select the appropriate gate in the flow cytometry. The results were presented as the percentage of cells in each phase.

### Cellular migration and invasion assay

Before assay, cells of every group were all cultured in medium without KnockOut™ Serum Replacement containing 0.1% bovine serum albumin (BSA) to pre-starve for 18h in migration assay or for 24h in invasion assay. Then cells were harvested and suspended at a certain concentration (2×10^6^/ml for migration assay and 4×10^6^/ml for invasion assay) in medium without Serum Replacement containing 0.1% BSA. A total of 200μl suspension was added into the upper chamber (Corning Costar, Cambridge, MA, USA) coated with 10μg Matrigel™ Basement Membrane Matrix (BD Biosciences) per well (for invasion assay) or uncoated (for migration assay). After 1h, the chambers were transferred to wells containing 600μl medium with 15% Serum Replacement and the incubation was conducted for 24 h. After removing cells and matrigel on the upper surface of the membrane with a cotton bud and 15min staining with 0.1% crystal violet, cell numbers on the underside were determined under a microscope. Five randomly selected fields were counted per insert. Cell invasion was evaluated with a Cell Invasion Assay Kit (ECM550, Millipore, MA, USA).

### Cellular senescence assay

Cellular senescence assay was performed with a senescence β-Galactosidase staining kit (C0602, Beyotime, China) according to the manufacturer’s instructions. β-galactosidase-positive staining cells was detected under a light microscope and five randomly selected fields were counted.

### Cellular colony formation assay

Cells were plated into 12-well plates at a density of 1x10^3^ cells per well and incubated for 15 days. Then, the cells were fixed with Methanol and stained with Giemsa (Sigma-Aldrich). The number of growing colonies was counted under an inverted light microscope.

### RNA isolation, RNA-sequencing and computational analysis

After culture in different FA level for 6 months, Total RNA of QSG-7701 cells were purified with an RNeasy Micro kit (74004, QIAGEN). Then, the Illumina PE Flow Cell v3-HS was used. The arrays were scanned with the Agilent Bioanalyser 2100 (Agilent Technologies, CA, USA). The sequencing process was controlled by the data collection software (Illumina, CA, USA) with Illumina Hiseq2500 platform, which possessed dynamic analysis capabilities. Each sample provided >5 G of original RNA-seq data with sequence reads > 90 nt and Q20 > 90%. RNA-sequencing assay and data analysis were entrusted to Shanghai Biotechnology Corporation (China). Briefly, raw data were aligned to the human reference genome hg19 using the Top Hat software. BAM-files were sorted and indexed using the Samtools software package. The RNA sequences of the three samples were analyzed by genomic mapping, gene expression analysis, differential gene analysis, lncRNA analysis and gene ontology (GO) and pathway analysis. GO analysis was applied to analyze the function of the differentially expressed genes according to the key functional classification of genes in the NCBI. Important pathways containing the differentially expressed genes were also performed by the Kyoto Encyclopedia of genes and Genomes (KEGG). The gene expression levels were analyzed based on the FPKM (Fragments per Kilobase of exon model per Million mapped reads) method using the Cufflinks software. The threshold used to screen up regulated or down regulated mRNA was a fold change ≥ 1.5 and a p-value ≤ 0.05.

### Reverse transcription (RT) reaction and quantitative real-time (Q-RT) PCR

RT reaction was performed with an M-MLV Reverse Transcriptase kit (Life Technologies). Q-RT PCR was performed by SYBR Green detection with a StepOne™ Plus Real-Time PCR System (Life Technologies). The expression of genes was normalized using the 2^-ΔΔCT^ method relative to β-actin endogenous control. All assays were performed in duplicate and data were analyzed by the StepOne™ Software (version 2.1, Life Technologies). All primers are listed in Supplementary Table 1.

### Transient and stable transfection

Transfection was performed using a Lipofectamine™ 3000 kit (Life Technologies) according to the manufacturer’s instructions. Different groups of cells were plated into 12-well plates (1×10^5^ cells/well) on day before transfection. Then, the siRNA for LCN2 and negative control (NC) were transfected into No FA cells respectively. Meanwhile, the pcDNA3.1-LCN2 and empty plasmid (pcDNA3.1) were transfected into High FA cells respectively. Stable transfectants (QSG-7701 cells with High FA) of pcDNA3.1-LCN2 or empty pcDNA3.1 were selected with G418 (Sigma-Aldrich) resistance (1000μg/ml followed by 500μg/ml). Finally, transient and stable transfectants were all validated by quantitative real-time PCR and western blot.

### Western blot

Cells were treated as indicated and the harvested cells were washed three times and lysed in RIPA lysis Buffer (R0278, Sigma-Aldrich). Lysates were incubated on ice for 10 min and then centrifuged at 8,000×g to remove cellular debris. The concentration of the extracted proteins was measured with a BCA protein assay kit (P0011, Beyotime, China). Forty to fifty micrograms of protein was used for Western transfer and immunobloting. Ponceau S staining of membranes verified proper transfer of proteins to nitrocellulose membranes. Primary antibodies were used (anti-LCN2. 1:300; anti-β-actin, 1:5000). A HRP-conjugated secondary antibody (1:10,000) was incubated for 2 h at room temperature, and the corresponding band was revealed using the DAB method (Sigma-Aldrich). As control for equal loading of the samples, the membranes were re-probed with β-actin.

### Hepatic mRNA level assay

Total RNA isolated from hepatic tissues was used for cDNA synthesis with an M-MLV Reverse Transcriptase kit. The hepatic mRNA levels of p53, LCN2, and β-actin were evaluated by semi-quantitative RT-PCR in the StepOne™ Real-Time PCR System according to follow parameters: 40 cycles of 40 min at 60°C, 3 min at 95° C, 1 min at 60° C and 20 seconds at 72°C. The PCR products were separated with 2% agarose gels. The signals of p53 and LCN2 were normalized against the signal of β-actin to analyze mRNA level. All primers are listed in Supplementary Table 1.

### Bisulfite genomic sequencing assay

The bisulfite genomic sequencing was performed to detect the methylation status of LCN2 promoter by Shanghai Ying Biotech Company. The PCR mixture was prepared as follows: 2X Master Mix (25 μl), Mg^2+^ (4 μl), F (10 μM, 1 μl), R (10 μM, 1 μl), and ddH_2_O to 50 μl. The PCR reaction was as follows: 95°C for 10 min; 95°C for 30 s, Tm for 30 s, 72°C for 30 s (35 cycles), and 72°C for 10 min. All primers are listed in Supplementary Table 1.

### Chromatin immunoprecipitation (ChIP) assay

ChIP assay was performed with an EZ-ChIP chromatin immunoprecipitation (ChIP) assay kit (17-295, Millipore) according to the manufacturer’s recommended procedure. The monoclonal antibodies for H3K9Me2 and nonspecific immunoglobulin G (56834, Sigma-Aldrich) were used for each sample. The amplified PCR products were electrophoresed with a 2% agarose gel.

### Nude mouse tumor formation assay (xenografts)

To examine the effect of FA on cell tumor formation *in vivo*, a total of 15 nude mice were randomly divided into three groups feeding with different amino acid-defined diet as follows: FA-deficiency nude mouse group (FD group, 0 mg folate/kg diet), FA-supplementation nude mouse group (FS group, 8 mg folate/kg diet), and FA-normal nude mouse group (FN group, 2 mg folate/kg diet). Then, different groups of cells (1×10^6^ cells/mouse) were separately injected subcutaneously into the left flanks of corresponding mice (High FA cells to FS group mice, Normal FA cells to FN group mice and Low FA cells to FD group mice). All mice were sacrificed to observe the development of tumor growth after 4-5 weeks after treatment. The tumor size of each group was measured at the same time of every week and the volume was calculated with the formula 1/2 L1 (L2)^2^, where L1 and L2 represented the long and short axes of the tumor, respectively. To examine the effect of the LCN2 on tumor formation *in vivo*, another 20 nude mice were randomly assigned to four groups as follows: Low FA cells to FD group mice, Normal FA cells to FN group mice, High FA cells (stable transfectants of pcDNA3.1) to FS group mice and High FA cells (stable transfectants of pcDNA3.1-LCN2) to FS group mice.

### HBx transgenic mouse tumor formation assay

To examine the chemoprevention effect of FA on HBx-induced hepatocarcinogenesis, a total of 15 HBx transgenic mice were also randomly divided into three groups feeding with different amino acid-defined diet as follows: FA-deficiency HBx mouse group (FD-HBx group, 0 mg folate/kg diet), FA-supplementation HBx mouse group (FS-HBx group, 8 mg folate/kg diet), and FA-normal HBx mouse group (FN-HBx group, 2 mg folate/kg diet).

### Statistical analysis

Data are presented as the means ± standard deviation. The data for two groups were not suitable for the Chi-square test; therefore, the Wilcoxon signed-rank test was used for the quantitative analysis of HCC tissues compared with adjacent tissues. The Mann-Whitney U test was used for two independent samples. One-way analysis of variance was performed for the quantitative analysis of groups. The data obtained from multiple time points were analyzed using repeated measurement data in the groups. Mauchly’s test showed that p<0.001. The results were revised using the Greenhouse-Geisser correction. The statistical analysis was performed using SPSS (version 18.0, IBM, NY, USA). A two-sided significance level of 0.001 was used for all statistical analyses.

## SUPPLEMENTARY MATERIALS FIGURE AND TABLE


