# Supplementary material for: Folic acid supplementation acts as a chemopreventive factor in tumorigenesis of hepatocellular carcinoma by inducing H3K9Me2-dependent transcriptional repression of LCN2
Source: Oncotarget. 2021 Feb 16;12(4):366–78. doi: 10.18632/oncotarget.27136 (PMC7899549; doi:10.18632/oncotarget.27136)
Supplement: Supplementary file 1 [file oncotarget-12-366-s001.pdf]

Folic acid supplementation acts as a chemopreventive factor in tumorigenesis of hepatocellular carcinoma by inducing H3K9Me2-dependent transcriptional repression of LCN2

SUPPLEMENTARY MATERIALS

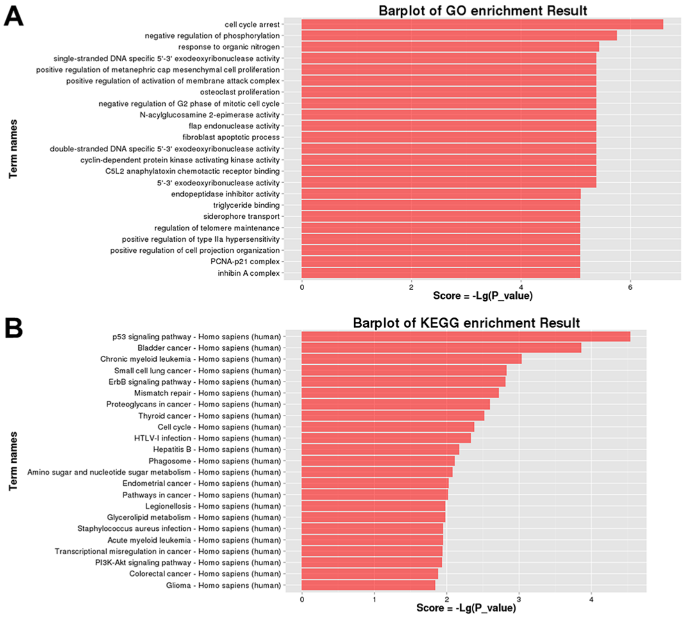

Supplementary Figure 1: Gene ontology (GO) and pathway analysis (KEGG) on data of RNA-sequencing assay. (A) GO analysis was applied to analyze the function of the differentially expressed genes according to the key functional classification of genes in the NCBI. **(B)** Important pathways containing the differentially expressed genes were also performed by the Kyoto Encyclopedia of genes and Genomes (KEGG).

Supplementary Table 1: Primers and oligonucleotide sequences enrolled in this study

Description	Name	Sequence
Primers for quantitative real-time PCR	CD133-human-real-F	5' ACGGAAGTCAGCTCCCATCA 3'
	CD133-human-real-R	5' GGCTCTCCAGATCGGTTCTG 3'
	OCT4-human-real-F	5' TTGAGGCTCTGCAGCTTAG 3'
	OCT4-human-real-R	5' GCCGGTTACAGAACCACAC 3'
	CK19-human-real-F	5' CCCGCGACTACAGCCACTACTAC 3'
	CK19-human-real-R	5' GTCGGCCTCCACGCTCATG 3'
	AFP-human-real-F	5' AGCTTGGTGGTGGATGAAAC 3'
	AFP-human-real-R	5' CCCTCTTCAGCAAAGCAGAC 3'
	CEBPA-human-real-F	5' CAAGAACAGCAACGAGTACCG 3'
	CEBPA-human-real-R	5' AGGCGGTCATTGTCACTGGT 3'
	ALB-human-real-F	5' CTGAGCAAAGGCAATCAACA 3'
	ALB-human-real-R	5' CACAGTCTGCTGAGGTTGGA 3'
	CK18-human-real-F	5' GAGCTGCTCCATCTGTAGGG 3'
	CK18-human-real-R	5' CACAGTCTGCTGAGGTTGGA 3'
	E-cadherin-human-real-F	5' GCCCCATCAGGCCTCCGTTT 3'
	E-cadherin-human-real-R	5' ACCTTGCCTTCTTTGTCTTTGTTGGA 3'
	Snail-human-real-F	5' TGCGTCTGCGGAACCTG 3'
	Snail-human-real-R	5' GGACTCTTGGTGCTTGTGGA 3'
	CK8-human-real-F	5' TCATAGACAAGGTACGGTTCC 3'
	CK8-human-real-R	5' GCCTAAGGTTGTTGATGTAGC 3'
	Slug-human-real-F	*5' CCTGGTTGCTTCAAGGACAC 3'*
	Slug-human-real-R	*5' TCCATGCTCTTGCAGCTCTC 3'*
	CDC20-human-real-F	*5' CGCCAGAGGGTTATCAGAACAGA 3'*
	CDC20-human-real-R	*5' TTCCCAGAACTCCAATCCACAAGG 3'*
	TNFRSF10D-human-real-F	*5' GAGATGTGCCGGACGTGTAG 3'*
	TNFRSF10D-human-real-R	*5' CTATGATGATAAGGTAGTGATAGGGAG 3'*
	MYC-human-real-F	*5' GGGGCTTTATCTAACTCGCTGTA 3'*
	MYC-human-real-R	*5' TATGGGCAAAGTTTCGTGGAT 3'*
	TP53INP1-human-real-F	*5' CACGGGCACAGAAGTGGAAG 3'*
	TP53INP1-human-real-R	*5' GGTGGCAATCCCTGGTAAGA 3'*
	ZMAT3-human-real-F	*5' TCAGCAGGTCCTTACTTCAA 3'*
	ZMAT3-human-real-R	*5' CTATACATATCCCAGATTCTCCA 3'*
	CDKN1A-human-real-F	*5' TCCTGTGGGCGGATTAG 3'*
	CDKN1A-human-real-R	*5' GTCACTGTCTTGTACCCATGTG 3'*
	LCN2-humam-real-F	*5' TGAGCACCAACTACAACCAGCAT 3'*
	LCN2-human-real-R	*5' CGATTGGGACAGGGAAGACG 3'*
	LCN2-mouse-real-F	*5' CCAGTTCGCCATGGTATTTTTC 3'*
	LCN2-mouse-real-R	*5' CACACTCACCACCCATTCAGTT 3'*

(Continued)

Description	Name	Sequence
	GDF15-human-real-F	*5' TTGCGGAAACGCTACGAG 3'*
	GDF15-human-real-R	*5' GGGACAGCCGGAACAGAG 3'*
	RTEL1-human-real-F	*5' ACGGACATCCCAAAGATTATTTACGC 3'*
	RTEL1-human-real-R	*5' GTTGTTGTAGAAATGACAGGAGCGACTT 3'*
	SESN1-human-real-F	*5' TCCAACATTTCGTGTCCAGG 3'*
	SESN1-human-real-R	*5' TAGTTCCAAATTGCCCGTCT 3'*
	CCNG1-human-real-F	*5' TAGTCTAACTCAGTTCTTTGGCTTTG 3'*
	CCNG1-human-real-R	*5' ATGGGACATTCCTTTCCTCTTC 3'*
	FPGS-human-real-F	*5' CCCCGAGGTTCGAGTCTTGCTC 3'*
	FPGS-human-real-R	*5' TGAAGTTCTGTTGGTCTGCGTTGC 3'*
	CDC45-human-real-F	*5' AGAAAGGAAGGCTGGGAACTAAG 3'*
	CDC45-human-real-R	*5' CACGTCCGAGGCCACGAAGA 3'*
	GAMT-human-real-F	*5' CCCACCCTGCCTGACGGTCACTTT 3'*
	GAMT-human-real-R	*5' GGGCACCTGCGTCTCCTCAAACAT 3'*
	DHFR-human-real-F	*5' TTCGCTAAACTGCATCGTCG 3'*
	DHFR-human-real-R	*5' GGTCTTCTTACCCATAATCACCAG 3'*
	RRM2-human-real-F	*5' AACTTGGTGGAGCGATTTAG 3'*
	RRM2-human-real-R	*5' CATAGGTAGCCTCTTTGTCCC 3'*
	P53-mouse-real-F	*5' AGCTTT GAGGTTCGTGTTTG 3'*
	P53-mouse-real-F	*5' GGAACATCTCGAAGCGTTTA 3'*
	GAPDH-human-real-F	*5' CTCTCTGCTCCTCCTGTTCG 3'*
	GAPDH-human-real-R	*5' ACGACCAAATCCGTTGACTC 3'*
	β-Actin-human-real-F	*5' TGTGTTGGCGTACAGGTCTTTG 3'*
	β-Actin-human-real-R	*5' GGGAAATCGTGCGTGACATTAAG 3'*
	β-Actin-mouse-real-F	*5' GCACCACACCTTCTACAATGAG 3'*
	β-Actin-mouse-real-R	*5' ACAGCCTGGATGGCTACGT 3'*
	U6-human-real-F	*5' CGCTTCACGAATTTGCGTGTCAT 3'*
	U6-human-real-R	*5' GCTTCGGCAGCACATATACTAAAAT 3'*
Primers for clone of LCN2	*LCN2-clone-F*	*5' GGGGTACCCCATGCCCCTAGGTCTCCTGTGGCT 3'*
	LCN2-clone-R	*5' CGGAATTCCGTCAGCCGTCGATACACTGGTCG 3'*
Primers for BSP	*Methy-LCN2-F*	*5' AAGTGTTGGAGTTATAGGYGAGAG 3'*
	Methy-LCN2-R	*5' ACCTTAAAAAATATCCTCTACCAAAC 3'*
Primer for identification of HBx positive mice	*Produce 1-F*	*5' CTCCAGATGGCAAACATACGCAAG 3'*
	Produce 1-R	*5' CAGGATCCAGTTGGCAGCACAG 3'*
	Produce 2-F	*5' CAAACTGATGACCATACGTGAAGAC 3'*
	Produce 2-R	*5' CCAAGGTCTTACATAAGAGGACTC 3'*
siRNA of LCN2	*LCN2-sense*	*5' CCACCAUCUAUGAGCUGAATT 3'*
	LCN2-anti-sense	*5' UUCAGCUCAUAGAUGGUGGTT 3'*
	NC-sense	*5' UUCUCCGAACGUGUCACGUTT 3'*
	NC-anti-sense	*5' ACGUGACACGUUCGGAGAATT 3'*
